# Ambiguity tolerance and resting-state functional connectivity: A preregistered conceptual replication in a Japanese sample

**DOI:** 10.1016/j.ynirp.2026.100376

**Published:** 2026-06-22

**Authors:** Jimpei Hitsuwari

**Affiliations:** a Experimental Psychology Unit, Faculty of Humanities and Social Sciences, Helmut Schmidt University, Germany; b Japan Society for the Promotion of Science, Japan

**Keywords:** Ambiguity tolerance, Resting-state functional connectivity, Multidimensional attitude toward ambiguity scale, Neural correlate, Conceptual replication

## Abstract

**Objective:**

To examine how three dimensions of the Multidimensional Attitude toward Ambiguity Scale (MAAS)—Discomfort with Ambiguity, Absolutism, and Need for Complexity—relate to resting-state functional connectivity, conceptually replicating and extending the work of Liu et al. (2023) in a Japanese sample. Liu et al. (2023) reported that higher ambiguity tolerance was associated with stronger connectivity in integration and control networks, whereas lower ambiguity tolerance was associated with stronger connectivity in threat- and error-monitoring circuits. Of the three MAAS dimensions, Need for Complexity was the one most closely aligned with their measure.

**Methods:**

Thirty-nine participants underwent resting-state MRI and completed the MAAS. Region-of-interest (ROI)-to-ROI analyses were used to test the associations between each MAAS dimension and its hypothesized connectivity pair, controlling for age, sex, and head motion.

**Results:**

No MAAS dimension was significantly associated with its corresponding connectivity pair. Effect sizes were negligible, although the Need for Complexity showed a small zero-order correlation with inferior parietal lobule–middle cingulate cortex connectivity.

**Conclusions:**

These findings contrast with earlier reports using unidimensional measures, suggesting that previously observed neural correlates may not map directly onto specific MAAS dimensions. Larger, well-powered, cross-cultural studies are needed.

## Highlights

• Linked three ambiguity-tolerance dimensions to resting-state connectivity.
• Preregistered conceptual replication of Liu et al. (2023) in a Japanese sample.
• No ambiguity dimension was significantly linked to its hypothesized pair.
• Need for Complexity, closest to the MSTAT-II, showed a small IPL–MCC correlation.
• Findings highlight the need for larger, well-powered neuroimaging samples.

## 1 Introduction

Ambiguity tolerance, the tendency to perceive ambiguous situations as desirable or threatening, has been a topic of psychological inquiry since Frenkel-Brunswik (1949) introduced the concept. A recent scientometric analysis showed substantial growth in this research area, with nearly 70% of the publications emerging in the past decade and expanding into medical education, career development, entrepreneurship, and political psychology (Rubiales-Núñez et al., 2024).

Despite this growth, neuroimaging studies examining the neural correlates of ambiguity tolerance are scarce. Liu et al. (2023) investigated resting-state functional connectivity in a Chinese sample (N = 315) using the Multiple Stimulus Types Ambiguity Tolerance Scale-II (MSTAT-II; McLain, 2009), a unidimensional measure that conceptualizes ambiguity tolerance as a continuum ranging from aversion to attraction to complex, unfamiliar, and insoluble stimuli. They reported that higher ambiguity tolerance was associated with stronger connectivity within the integration and control networks (inferior parietal lobule [IPL]–middle frontal gyrus and middle cingulate cortex [MCC]), whereas lower tolerance was linked to stronger connectivity in threat and error-monitoring circuits (orbitofrontal cortex and anterior cingulate cortex [ACC]). These findings reflect a balance between neural systems supporting flexible information integration and those treating ambiguity as a threat. Other studies have linked ambiguity tolerance with gray matter density in the right inferior frontal gyrus, supporting flexible reappraisal and inhibition (Tong et al., 2023), and to differential activation patterns in visual association areas during ambiguous stimulus processing (Mazhirina et al., 2020). However, Tanaka et al. (2015) found no association between self-reported ambiguity intolerance and gray matter volume, highlighting the inconsistencies in the literature.

Dimensionality is a critical issue in ambiguity tolerance research. Historically, the construct has suffered from conceptual fragmentation, grouping emotional discomfort, black-and-white thinking, and novelty-seeking under a single label (Lauriola et al., 2016). To address this, Lauriola et al. (2016) developed the Multidimensional Attitude toward Ambiguity Scale (MAAS), identifying three robust factors: Discomfort with Ambiguity (DA), reflecting anxiety in ambiguous situations; Absolutism (AB), the tendency toward dichotomous terms; and the Need for Complexity (NC), a preference for complexity, multiplicity, and novelty.^1^ The MAAS-based research has demonstrated distinct associations in each dimension. For instance, DA and NC differentially predict attitudes toward artificial intelligence (Hitsuwari and Takano, 2025), AB predicts reduced analytical thinking (van Zyl, 2021), and the haiku intervention selectively reduces AB, leaving DA and NC unchanged (Hitsuwari and Nomura, 2023a).

Given the multidimensional nature of ambiguity tolerance, earlier neuroimaging findings based on unidimensional measures may have obscured dimension-specific neural correlates. The MAAS was developed from a pool of 133 items taken from seven existing ambiguity tolerance scales, including the MSTAT-I (Lauriola et al., 2016); the MSTAT-II used by Liu et al. (2023) is a later revision of the MSTAT-I that shares the same conceptual definition (McLain, 2009). The MAAS distributes this content across three empirically derived dimensions. Among them, the Need for Complexity dimension corresponds most closely to the content captured by the MSTAT family, whereas Discomfort with Ambiguity and Absolutism draw more heavily on other scales. Thus, its items are theoretically connected to the content of prior unidimensional measures while distributing that content across three empirically derived dimensions. This study conceptually replicated and extended the work of Liu et al. (2023) by examining how the three MAAS dimensions relate to resting-state functional connectivity in a Japanese sample. In doing so, the present study makes three contributions beyond direct replication. First, it extends previous research by examining the neural correlates of ambiguity tolerance using a multidimensional measure. Second, it investigates these associations in a Japanese sample, a population that has not been examined previously. Third, it enables an empirical assessment of which specific dimension(s) of ambiguity tolerance, if any, account for previously reported neural associations. Accordingly, three preregistered hypotheses were formulated:

H1 (Discomfort with Ambiguity): Higher DA scores are associated with stronger functional connectivity between the amygdala and anterior insula. Because DA captures the affective discomfort component of ambiguity attitudes, and the amygdala and anterior insula were selected by Liu et al. (2023) to index salience and threat anticipation, greater discomfort is predicted to accompany stronger connectivity in this circuit.

H2 (Absolutism): Higher AB scores are associated with stronger functional connectivity between the left OFC and ACC, a circuit implicated in ambiguity-related warning signals.

H3 (Need for Complexity): Higher NC scores are positively associated with connectivity between the left IPL and the left MFG/MCC, which has been associated with the flexible integration of ambiguous information.

## 2 Method

Ethical approval was obtained from the Graduate School of Education, Kyoto University (CPE-510), and the Institute for the Future of Human Society MRI Research Facility, Kyoto University (22-003). All procedures were conducted in accordance with the Declaration of Helsinki and in accordance with the institutional guidelines. Written informed consent was obtained from all participants before their participation. The resting-state functional MRI data analyzed here were acquired in the same scanning session, and from the same participants, as the haiku-appreciation task reported by Hitsuwari and Nomura (2025a), but were not analyzed or reported in that study. They were analyzed here for the first time. The specific hypotheses and analyses reported here are independent of those of that study. The study was preregistered on the Open Science Framework (https://osf.io/vhbq2). The analysis scripts and behavioral data are publicly available at https://osf.io/84ev5.

### 2.1 Participants

Participants were recruited through an electronic bulletin board at Kyoto University, and data were collected between September and December 2022. Eligibility was restricted to right-handed, native Japanese-speaking, healthy adults with no neurological or psychiatric disorders and no MRI contraindications; sex was not a recruitment criterion. Forty-four adults were scanned, of whom five were excluded because of incomplete or unusable data, leaving 39 participants (M_age_ = 21.69, SD = 1.64; range = 18–25; 24 men and 15 women). Participants received 2500 JPY as compensation. All the participants had normal or corrected-to-normal vision.

### 2.2 Questionnaires

#### 2.2.1 Multidimensional Attitude toward Ambiguity Scale (MAAS)

Ambiguity tolerance was assessed using the Japanese version of the MAAS (Hitsuwari and Nomura, 2021). On the day of the experiment, participants completed the MAAS via email before the MRI session. The 21-item scale comprises three seven-item subscales: DA (negative emotional reactions to ambiguity), AB (rigid, dichotomous thinking), and NC (desire for novel and complex information). Participants rated each item on a 7-point Likert scale ranging from 1 (strongly disagree) to 7 (strongly agree). Mean subscale scores were calculated.

#### 2.2.2 Other measures

Additional questionnaires were administered as part of a larger study protocol: the shortened Japanese versions of the Plymouth Sensory Imagery Questionnaire (Hitsuwari and Nomura, 2023b), the Interpersonal Reactivity Index (Himichi et al., 2017), the Awe subscale of the Dispositional Positive Emotion Scales (Nomura et al., 2022), the Southampton Nostalgia Scale (Nagamine and Toyama, 2019), and the Big Five Scale (Namikawa et al., 2012), though not analyzed in the present study.

### 2.3 MRI acquisition

MRI images were acquired using a 3-T Siemens Verio scanner with a 32-channel head coil at the Institute for the Future of Human Society, Kyoto University. Resting-state functional images were acquired using a multiband echo-planar imaging sequence: multiband factor = 4, number of slices = 76, slice thickness = 2.0 mm; voxel size = 2 × 2 × 2 mm, flip angle = 80°, repetition time (TR) = 2000 ms; echo time (TE) = 43 ms, field of view = 192 mm, and acquisition matrix = 96 × 96. Each resting-state scan lasted 6 min and yielded 180 volumes. Participants kept their eyes open and fixed on a central crosshair.

High-resolution T1-weighted structural images were acquired using a magnetization-prepared rapid acquisition gradient echo (MPRAGE) sequence: number of slices = 208; slice thickness = 1 mm; voxel size = 1.0 × 1.0 × 1.0 mm^3^; flip angle = 9°; TR = 2250 ms; TE = 3.51 ms; field of view = 256 mm; acquisition matrix = 256 × 256.

### 2.4 Data analysis

#### 2.4.1 Pre-processing

Functional image pre-processing was performed using SPM25 in MATLAB R2024b. The first four volumes were discarded for T1 equilibration, leaving 176 vol per participant for analysis. Pre-processing steps included (1) slice timing correction with the middle slice as the reference, (2) motion realignment, (3) co-registration of functional images to the T1-weighted structural image, (4) normalization to the Montreal Neurological Institute (MNI) space using deformation fields derived from structural image segmentation, and (5) spatial smoothing with an 8-mm full-width at half-maximum (FWHM) Gaussian kernel.

#### 2.4.2 Functional connectivity analysis

Resting-state functional connectivity analysis was conducted using CONN toolbox version 22 (Whitfield-Gabrieli and Nieto-Castanon, 2012). Denoising was performed using the anatomical component-based noise correction method (aCompCor; Behzadi et al., 2007), regressing 16 principal components each from white matter and cerebrospinal fluid signals along with 12 motion parameters (six realignment parameters and their first-order derivatives). Data were bandpass-filtered (0.01–0.1 Hz) to remove low-frequency drift and high-frequency physiological noise, and then linearly detrended. Rather than regressing the mean white-matter signal, this approach extracts principal components that estimate the structured physiological and motion-related noise shared with the gray-matter signal. Although white-matter BOLD signals are not purely artifactual and may carry neural-activity-related information (Gawryluk et al., 2014; Gore et al., 2019, 2026), the regions of interest in the present study were defined to target gray-matter regions following the coordinates reported by Liu et al. (2023). Therefore, the noise components and the cortico-cortical connections under investigation derive from largely distinct tissue compartments.

#### 2.4.3 Regions of interest

Based on Liu et al. (2023), spherical regions of interest (ROIs; 6-mm radius) were defined following MNI coordinates: bilateral amygdala (left: −22, −6, −14; right: 26, −2, −16), bilateral anterior insula (left: −32, 23, 4 and −26, 26, 4; right: 35, 24, 0 and 39, 18, 2), left OFC (−30, 54, −4; −38, 50, −12; −28, 52, −14), bilateral ACC (6, 22, 31 and 6, 30, 36), left IPL (−48, −39, 30), left MFG (−12, 33, 60), and MCC (3, −30, 36). For ROIs with multiple coordinate peaks, the signals were averaged across all the spheres to create a single bilateral or composite ROI. Although the specific coordinates were not listed in the preregistration, they were fully determined by the preregistered decision to adopt the coordinates reported by Liu et al. (2023) and thus involved no analytic discretion in their selection.

#### 2.4.4 Statistical analysis

Fisher's z-transformed correlation coefficients were computed for three ROI pairs corresponding to the following hypotheses: (H1) bilateral amygdala–bilateral anterior insula, (H2) left OFC–bilateral ACC, and (H3) left IPL–left MFG/MCC. Multiple regression was used to test the associations between MAAS subscale scores and functional connectivity, controlling for age, sex, and mean framewise displacement (FD) as a measure of head motion. The mean FD was included as a covariate of no interest to control for inter-individual differences in head motion, which are known to systematically bias functional connectivity estimates and co-vary with individual differences, thereby potentially confounding the analyses relating connectivity to person-level variables (Power et al., 2012). A Bonferroni-corrected threshold of *p* < .017 (.05/3) was used.

## 3 Results

Descriptive statistics are presented in Table 1. The sample consisted of 39 healthy young adults (24 male and 15 female) with a mean age of 21.7 years (SD = 1.6, range = 18–25 years). The mean frame-wise displacement was 0.153 mm (SD = 0.050), indicating low head motion throughout the scanning session. The MAAS subscales showed low intercorrelations, except for a moderate positive correlation between DA and NC (*r* = 0.42, *p* = .008).

**Table 1:** Descriptive statistics for demographic variables, MAAS subscales, and functional connectivity measures.

Variable	M	SD	Range
Age (years)	21.69	1.64	18–25
Discomfort with Ambiguity	4.88	0.83	3.00–6.57
Absolutism	2.93	0.85	1.29–4.71
Need for Complexity	4.37	1.1	1.86–6.71
Mean FD (mm)	0.15	0.05	0.08–0.27
FC Amygdala–AI (Fisher's z)	−0.02	0.18	—
FC OFC–ACC (Fisher's z)	0.21	0.21	—
FC IPL–MFG/MCC (Fisher's z)	−0.1	0.13	—

Associations between the three MAAS subscales and their hypothesized functional connectivity pairs were examined by controlling for age, sex, and mean framewise displacement. Contrary to the preregistered hypotheses, none of the predicted associations reached statistical significance.

For Hypothesis 1, Discomfort with Ambiguity was not significantly associated with connectivity between the bilateral amygdala and anterior insula (*β* = 0.003, *t* (34) = 0.07, *p* = .942). For Hypothesis 2, Absolutism showed no significant association with connectivity between the left OFC and ACC (*β* = −0.027, *t* (34) = −0.67, *p* = .509). For Hypothesis 3, Need for Complexity was not significantly associated with connectivity between the left IPL and the MFG/MCC (*β* = 0.019, *t* (34) = 0.93, *p* = .359).

**Fig. 1:**
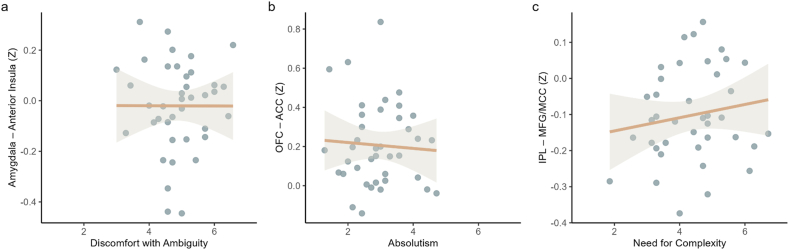
Associations between MAAS subscales and resting-state functional connectivity. (a) Discomfort with Ambiguity – amygdala–anterior insula. (b) Absolutism – OFC–ACC. (c) Need for Complexity – IPL–MFG/MCC. *Note*. Shaded areas represent 95% confidence intervals.

All p-values were substantially above the Bonferroni-corrected threshold (*α* = .017), and the standardized effect sizes were negligible (all *|β|* < 0.03). Fig. 1 presents scatter plots for each MAAS subscale and its corresponding connectivity measures.

As a supplementary analysis, the Need for Complexity for each target region was examined separately. Neither IPL–MFG connectivity (*β* = 0.007, *t* (34) = 0.24, *p* = .810) nor IPL–MCC connectivity (*β* = 0.032, *t* (34) = 1.05, *p* = .301) was significant, confirming that averaging across regions did not mask potential effects. In an exploratory post hoc observation, the zero-order correlation between NC and IPL–MCC connectivity showed a small-to-medium effect size (*r* = 0.202, *p* = .217), although this was not statistically significant.

## 4 Discussion

This study aimed to conceptually replicate and extend Liu et al. (2023) by examining the association between the three dimensions of ambiguity tolerance and resting-state functional connectivity in a Japanese sample. Contrary to preregistered hypotheses, none of the hypothesized associations reached statistical significance. Discomfort with Ambiguity was unrelated to amygdala–anterior insula connectivity, Absolutism showed no relationship with OFC–ACC connectivity, and Need for Complexity was not associated with IPL–MFG/MCC connectivity. These null findings are consistent with those of Tanaka et al. (2015), who reported no structural correlates between ambiguity intolerance and brain structure.

Several methodological differences from Liu et al. (2023) may explain these divergent findings. First, Liu et al. employed seed-to-voxel whole-brain analyses, the sensitivity of which depended on a large sample size (N = 315), particularly for the weak, distributed brain–behavior effects typical of individual difference analyses (Cremers et al., 2017). Given the present sample (N = 39), I restricted testing to the connections identified by Liu et al. as significant and evaluated them using low-degree-of-freedom ROI-to-ROI regressions. This represents a deliberate adaptation to sample size rather than a procedural deviation, whereas Liu et al.'s whole-brain analysis was exploratory; the present analysis constitutes a more hypothesis-driven, methodologically independent test of the connections it identified. Although the ROIs were defined using the coordinates of Liu et al., subtle differences in the ROI construction may have influenced the connectivity estimates. Moreover, the regions and connections examined here are not specific to the functions invoked in the hypotheses; each participates in multiple cognitive processes, and functional interpretations should accordingly be read as motivating rationales drawn from prior work rather than as established attributions (Poldrack, 2006). Second, and more critically, ambiguity tolerance was operationalized differently: Liu et al. used a unidimensional construct, while I examined three MAAS dimensions. I chose not to compute the composite MAAS scores. The MAAS subscales were developed as conceptually and statistically distinct dimensions (Lauriola et al., 2016). Although Lauriola et al. proposed a bifactor structure in which a single total score reliably indexes a general ambiguity-avoiding attitude, they nonetheless recommend the use of subscale scores when investigators are interested in specific facets of ambiguity attitudes. Moreover, Lauriola et al. (2016) explicitly state that the MSTAT-I "shares strong content coverage with our NC factor" (p. 369); the MSTAT-II shares the same conceptual definition as the MSTAT-I (McLain, 2009). Aggregating NC with DA and AB into a composite would thus dilute, rather than enhance, the comparability with the measurements of Liu et al. From this perspective, the small-to-medium zero-order correlation between NC and IPL–MCC connectivity (r = 0.202) reported in my supplementary analysis, in the direction predicted by Liu et al. and approaching their estimated effect size, is broadly consistent with what one might expect under an underpowered conceptual replication rather than a clean disconfirmation. Third, the cultural differences between the samples may be relevant. Liu et al. studied Chinese participants, whereas the present sample comprised young Japanese adults. Although the MAAS demonstrates cross-cultural validity (Lauriola et al., 2016), the neural correlates of the ambiguity-tolerance dimensions may vary across cultural contexts (Hitsuwari and Nomura, 2021; Spector et al., 2001).

The near-zero amygdala–anterior insula connectivity (Fisher's z = −0.02) warrants comment. Similarly, Liu et al. (2023) found no significant resting-state connectivity for these regions, attributing this to their task-based origin in a task-free design similar to the present one. That a non-significant pair, even in their larger sample, showed little connectivity is therefore unsurprising, and the substantial between-participant variability (SD = 0.18) precludes reading this near-zero mean as evidence that these regions are unrelated to ambiguity tolerance.

Despite these null findings, this study makes a modest contribution to the literature. Preregistered replications, including null results, are essential to establish the robustness of scientific findings. The neuroimaging literature on ambiguity tolerance remains limited and inconsistent; some studies have reported significant associations (Liu et al., 2023; Tong et al., 2023), whereas others have not (Tanaka et al., 2015). Due to its modest sample size, the present study could not establish the neural substrates of ambiguity tolerance. Reported alongside the existing inconsistent findings, however, it contributes a preregistered null result that helps counteract the publication bias toward significant associations and provides a data point for future meta-analytic synthesis; collectively, this literature points to the need for well-powered, preregistered investigations to resolve the discrepancies. As discussed above, the supplementary observation that the NC–IPL–MCC correlation approached the effect size estimated by Liu et al. further suggests that larger samples may be required to reliably detect such associations.

### 4.1 Limitations and future directions

This study had several limitations. First, the small sample size (N = 39) limited the power to detect small effects, and near-zero confirmatory coefficients could not be distinguished from the true absence of association in these data alone. The results, therefore, indicate that the hypothesized associations were absent, but the effects of the magnitude reported by Liu et al. were unlikely to be detected here, consistent with the supplementary NC–IPL–MCC correlation, which approached that magnitude. Larger multisite samples are needed to detect such effects and would enable cross-cultural comparisons.

Second, the resting-state run was limited to 6 min (180 vol) because the data were obtained from a protocol optimized for a separate study (Hitsuwari and Nomura, 2025a). Although this duration is common, the reliability of the connectivity estimates, especially for individual differences, improves with longer acquisitions (Birn et al., 2013). Thus, the short run compounds the power limitation above, and the results are best read as non-detection under limited reliability and power rather than as evidence of absent associations.

Third, ambiguity tolerance was operationalized multidimensionally (MAAS), whereas Liu et al. used a unidimensional measure (MSTAT-II). This complicates direct comparison but is a deliberate design feature rather than a flaw, permitting a dimension-specific test that the original measure could not. No single subscale maps perfectly onto MSTAT-II, though Need for Complexity comes closest among the three.

Fourth, only the resting-state connectivity was examined. Ambiguity tolerance may be more readily expressed in tasks involving ambiguous stimuli; indeed, attitudes toward ambiguity interact with appreciation of the haiku (Hitsuwari and Nomura, 2025b), so associations that are undetectable at rest could emerge during such tasks. This binds the conclusions to intrinsic connectivity rather than undermining the resting-state analyses.

Finally, the young adult sample has limited generalizability. Intolerance of uncertainty, a construct closely related to ambiguity tolerance, differs with age across adulthood (Yu et al., 2025), so the present pattern may not extend to older populations, although this does not affect internal validity within the sample.

## 5 Conclusion

In conclusion, this study did not replicate the association between ambiguity tolerance and resting-state functional connectivity as reported by Liu et al. (2023). Although null findings may reflect a genuine absence of effects, methodological differences prevent definitive conclusions. The observed small-to-medium effect size for the NC–IPL–MCC association suggests that the neural correlates of ambiguity tolerance and brain connectivity warrant further investigation using larger samples.

## Ethical statement

Ethical approval was obtained from the Graduate School of Education, Kyoto University (CPE-510), and the Institute for the Future of Human Society MRI Research Facility, Kyoto University (22-003). All procedures were conducted in accordance with the Declaration of Helsinki and were in accordance with the institutional guidelines. Written informed consent was obtained from all participants before their participation. This study was preregistered on the Open Science Framework (https://osf.io/vhbq2).

## Declaration of generative AI in scientific writing

During the preparation of this work, the author used Claude Sonnet 4.5 (Anthropic) to assist with writing the MATLAB scripts for neuroimaging data analysis and drafting the manuscript in English. After using this tool, the author reviewed and edited the content as required and took full responsibility for the published article.

## Funding

This work was supported by the 10.13039/501100001691Japan Society for the Promotion of Science Overseas Research Fellowship and crowdfunding from an academist (https://academist-cf.com/fanclubs/358).

## Declaration of competing interest

The authors declare that they have no known competing financial interests or personal relationships that could have appeared to influence the work reported in this paper.

## Data Availability

Behavioral data and analysis scripts are publicly available in the Open Science Framework (https://osf.io/84ev5), with a persistent DOI assigned upon public release at acceptance. Raw MRI data are not publicly available because ethics approval and participant consent did not include provisions for the open release of imaging data; de-identified data are available from the author upon reasonable request and are subject to institutional approval.

## Footnotes

1 In the original MAAS, Absolutism is labeled “Moral Absolutism/Splitting” and Need for Complexity is labeled “Need for Complexity and Novelty.” I use the abbreviated terms throughout this paper for brevity.
